# Evaluation of Nucleation and Growth Kinetics of Li_3_PO_4_ Reactive Crystallization from Low-Concentration Lithium-Rich Brine

**DOI:** 10.3390/molecules31020392

**Published:** 2026-01-22

**Authors:** Jie Fan, Xiaoxiang He, Wanxia Ma, Chaoliang Zhu, Guowang Xu, Zhenghua He, Yifei Shi, Bo Li, Xiaochuan Deng

**Affiliations:** 1 Key Laboratory of Green and High-End Utilization of Salt Lake Resources, Qinghai Institute of Salt Lakes, Chinese Academy of Sciences, 18 Xinning Road, Xining 810001, China; fjie@isl.ac.cn (J.F.); hexiaoxiang22@mails.ucas.ac.cn (X.H.); mawanx@isl.ac.cn (W.M.); zhchl@isl.ac.cn (C.Z.); guowang_xu@isl.ac.cn (G.X.); hezhenghua@isl.ac.cn (Z.H.); shiyf@isl.ac.cn (Y.S.); 2 Qinghai Engineering and Technology Research Center of Comprehensive Utilization of Salt Lake Resources, Qinghai Institute of Salt Lakes, Chinese Academy of Sciences, 18 Xinning Road, Xining 810001, China; 3 University of Chinese Academy of Sciences, 19A Yuquan Road, Beijing 100049, China

**Keywords:** Li_3_PO_4_, lithium-rich brine, reactive crystallization, nucleation kinetics, growth kinetics

## Abstract

Li_3_PO_4_ is a promising raw material for the low-cost synthesis of high-performance LiFePO_4_. Reactive crystallization from low-concentration lithium-rich brine is a key process for the efficient preparation of high-quality Li_3_PO_4_ products. The effect of operating conditions (temperature/supersaturation/impurities/ultrasonic) on the induction time was investigated using a focused beam reflectance measurement. The evaluation of the primary nucleation, growth kinetics, and parameters for the extraction of Li_3_PO_4_ from low-concentration lithium-rich brine was conducted using an induction time method. The dominant mechanisms at different stages were inferred through online monitoring of the particle size distribution during the Li_3_PO_4_ crystallization process. Results show that induction time decreases with increasing operating conditions (temperature/supersaturation/ultrasonic frequency), indicating that their increases all promote nucleation. Impurities (NaCl/KCl) did not significantly affect the induction time, whereas Na_2_SO_4_ and Na_2_B_4_O_7_ significantly increased it, with Na_2_B_4_O_7_ showing the most notable effect. Classical nucleation theory was applied to determine kinetic parameters (nucleation activation energy/interfacial tension/contact angle/critical nucleus size/surface entropy factor). Results indicate that Li_3_PO_4_ mainly nucleates through heterogeneous nucleation, with a temperature increase weakening the role of heterogeneous nucleation. Fitted models indicate that Li_3_PO_4_ predominantly follows the secondary nucleation and spiral growth mechanism. Our findings are crucial for crystallization design and control in producing high-quality Li_3_PO_4_ from lithium-rich brines.

## 1. Introduction

Lithium phosphate (Li_3_PO_4_) is not only the preferred precursor for the cost-effective preparation of the high-performance lithium-ion battery cathode material LiFePO_4_ [1,2] but is also widely used in lithium-ion batteries [3], fluorescent materials [4], catalysis [5], and glass industries [6]. Driven by the rapid development of electric vehicles and energy storage sectors in recent years [7,8], the global demand for lithium salts has continued to rise, making the development of efficient lithium extraction technologies particularly urgent. As a crucial lithium resource, salt lake brine accounts for over 60% of global lithium reserves [9], with this proportion reaching as high as 71.9% in China [10]. The Dong−Taijinaier salt lake in China stands as one of the most representative salt lakes with the richest lithium resources and highest lithium content domestically [11]. From an economic perspective, the cost of lithium extraction from salt lakes is merely 30–50% of that from mineral ores [12]. Therefore, salt lake brine extraction technology has become the mainstream direction for lithium resource development.

Compared to lithium carbonate (K_sp_ = 2.5 × 10^−2^ at 298.15 K), the main lithium salt product from salt lakes, Li_3_PO_4_ (K_sp_ = 2.37 × 10^−11^ at 298.15 K), exhibits extremely low solubility, enabling effective precipitation from solutions at low lithium concentrations. This makes it a promising candidate for enhancing lithium recovery from salt lake brine. Liu et al. [13] achieved impurity purification and efficient lithium extraction in the lithium-rich solution through phosphate-selective precipitation, thereby solving the problem of poor lithium recovery under low-temperature and low-lithium-concentration conditions. Zhao et al. [14] demonstrated an 85.1% lithium recovery through ultrasonic-assisted Li_3_PO_4_ recovery from low-concentration lithium solutions. Tao et al. [15] achieved 85.56% lithium recovery using a high-temperature activation method from spent lithium iron phosphate cathode materials. Song et al. [16] developed a method to precipitate lithium using sodium phosphate from alkaline leachate, with the alkaline mother liquor being reusable. Sun et al. [17] prepared Li_3_PO_4_ with uniform particle size (5–8 μm) and over 90% yield using a hydrothermal method from lithium−tail liquid generated during salt lake lithium extraction, employing sodium phosphate as a precipitant. These studies demonstrate that the Li_3_PO_4_ precipitation method represents an economically efficient approach for lithium recovery from low-concentration lithium solutions.

Reactive crystallization is not only an efficient separation and purification technology but also a crucial process engineering approach. Liu et al. [18] achieved a lithium-recovery rate of 51.62% through low-temperature-induced precipitation using active Li_3_PO_4_ seed crystals at 303.15 K. The lithium-recovery rate using active Li_3_PO_4_ seed-crystal-induced precipitation matched that of high-temperature precipitation at 363.15 K, but with a significantly reduced reaction time. Li et al. [19] systematically investigated the effects of multiple process parameters on the Li_3_PO_4_ yield and particle size during reactive crystallization, achieving a lithium recovery rate of 96.85% and particle size of 18.70 μm through optimized processes. Hu et al. [20] employed coprecipitation to explore reaction conditions for preparing ultrafine Li_3_PO_4_ in a turbulent loop reactor, obtaining a narrow particle size distribution (D_50_ = 3.25 μm) and uniformly dispersed ultrafine lithium phosphate powder. Therefore, crystallization process control aimed at obtaining high-quality products holds significant importance.

The induction time method is a relatively simple, intuitive, and information-rich experimental approach for studying reactive crystallization kinetics [21,22]. Its core lies in precisely controlling supersaturation and accurately measuring the “time of the first crystal appearance.” By analyzing the relationship between the induction time and supersaturation, it can provide profound insights into the mechanisms of reactive crystallization kinetics. Current induction time measurement methods include the focused beam reflectance measurement (FBRM) [23,24], particle vision and measurement (PVM) [25], turbidity method [26], laser method [22], and conductivity method. Among these, FBRM can detect particles as small as submicron sizes (typically down to 0.5 μm, but still significantly larger than the size of the nucleus). Therefore, the “induction time” measured by these instruments is more accurately defined as the time required for the first stable atomic nucleus to form and subsequently grow to a detectable size. Even so, it can still capture nucleation signals in the early stages when the crystals are much smaller than visible to the naked eye or undetectable using the turbidity method. The induction time detected using this method is closer to the true “first nucleation” time with minimal delay error. Due to its high sampling frequency (every 2 s) and rapid response, FBRM is particularly suitable for studying reactive crystallization processes [27].

Nucleation and growth are the two most critical steps in reactive crystallization and also a key aspect of process control. Operational parameters (temperature, supersaturation, agitation rate, impurities, field effects, etc.) exert significant effects on crystallization processes. Slight operational fluctuations can induce changes in nucleation and growth, substantially affecting product properties. For instance, excessively high supersaturation leads to rapid nucleation and the excessive formation of fine crystals, while rapid crystal growth may encapsulate impurities or mother liquor within the lattice, compromising product purity. The in-depth exploration of nucleation and growth mechanisms in reactive crystallization serves as the foundation for industrial crystallization process control. According to nucleation and growth theory, the induction time under different supersaturation levels can be fitted to obtain the nucleation, growth mechanisms, and kinetic parameters. Emmanuel et al. [28] simultaneously injected LiCl and Na_3_PO_4_ reaction precursors into microchannels, where precipitation reactions occurred under concentration gradients. By monitoring, they studied the growth kinetics of individual precipitated particles and prepared orthorhombic Li_3_PO_4_ polycrystalline aggregates with flow-controlled particle sizes. Emmanuel et al. [29] established a power law resembling the law of mass action to describe Li_3_PO_4_ nucleation under stoichiometric reactant concentrations by monitoring the turbidity of well-stirred reaction mixtures at different concentrations and temperatures. Recently, ultrasound has emerged as an effective crystallization-strengthening technique capable of producing products with more uniform particle sizes and higher purity. This is of great significance for meeting the high-quality requirements of downstream industries. Based on previous studies on ultrasound-enhanced crystallization processes [30], this work investigates the influence of ultrasound on the nucleation process of Li_3_PO_4_ by utilizing ultrasonic micro-mixing and cavitation-induced secondary nucleation effects.

The Qinghai−Tibet Plateau in China is home to numerous salt lakes rich in lithium resources. Currently, the Li_2_CO_3_-production process represented by the Dong−Taijinaier salt lake requires concentrating lithium ions in brine to above 20 g·L^−1^ before precipitation. Compared to Li_3_PO_4_, Li_2_CO_3_ exhibits higher solubility (13.3 g·L^−1^ at 293.15 K), resulting in the existing process not only consuming significant energy but also achieving a lithium-recovery rate below 80%. Therefore, studying direct lithium extraction via phosphate precipitation from the low-concentration lithium-rich brine ([Li^+^] = 8.9 g·L^−1^) of the Dong−Taijinaier salt lake is crucial for improving resource utilization and reducing energy consumption. However, coexisting impurities such as NaCl, KCl, Na_2_SO_4_, and Na_2_B_4_O_7_ in lithium-rich brine not only lower lithium-recovery rates but also impair Li_3_PO_4_-crystallization behavior, resulting in poor product quality and process instability. Understanding how these impurities affect Li_3_PO_4_ crystallization is essential for developing impurity-removal strategies and optimizing crystallization processes [31]. Our previous work investigated the thermodynamics of Li_3_PO_4_ dissolution in mixed salt solutions (NaCl−KCl−Na_2_SO_4_−Na_2_B_4_O_7_) [32], with experimental and simulations of Li_3_PO_4_ reactive crystallization from low-concentration lithium−rich brine [31]. Based on the aforementioned research, this study systematically investigated the effects of temperature, supersaturation, impurities, and the ultrasonic method on the induction time through FBRM measurements. We evaluated the primary nucleation and growth kinetics of Li_3_PO_4_ crystallization in low-concentration lithium-rich brine using the induction time method and regressed the kinetic parameters. Additionally, online monitoring of particle size distribution changes during Li_3_PO_4_ reactive crystallization clarifies the nucleation and growth steps. These findings will provide kinetic guidance for low-cost and efficient lithium recovery from salt lake brine, enabling the more efficient and sustainable industrial production of high-quality Li_3_PO_4_.

## 2. Theory

### 2.1. Supersaturation and Induction Time

Supersaturation refers to a state where the solute concentration in a solution exceeds its equilibrium solubility under certain conditions. It is also commonly expressed as a concentration ratio (*S* = *c*/c*, where *c* is the solute concentration and c* is the equilibrium solubility of the solute) [33]. This study was conducted in a lithium-rich brine system (a high-ionic-strength solution), where concentration-based supersaturation would be overestimated. Due to the lack of data, such as activity coefficients, for this specific reaction system, all nucleation and growth kinetic parameters reported in this study should be considered as apparent parameters. These parameters have comprehensively incorporated the effects of solution non-ideality and represent practical approximations under high-ionic-strength conditions. However, all kinetic data comparisons in this study are based on this common standard to ensure the comparability of relative trends.

The crystallization of a solution can be regarded as two processes: nucleation and crystal growth. However, once crystallization begins, nucleation and crystal growth will cooperate to consume the supersaturation. The relationship between nucleation and crystal growth determines the particle size and particle size distribution of the product, making it a crucial aspect of industrial crystallization processes.

The induction time refers to the time delay between establishing a supersaturated state and the occurrence of a detectable stable nucleation event. It is significantly influenced by factors such as supersaturation, the presence of impurities, the agitation state, and viscosity. According to Mullin’s statement [34], this process can be considered composed of several parts or stages: *t*_r_, *t*_n_, and *t*_g_. *t*_r_ represents the relaxation time required for the system to achieve a quasi-stationary distribution of clusters. For large solute molecules like polymers or protein molecules, the relaxation time is significant. It can be neglected in low-viscosity aqueous solutions. *t*_n_ denotes the nucleation time required to form critical nuclei. *t*_g_ refers to the growth time needed for crystals to grow from critical size to detectable crystal dimensions. Therefore, by neglecting the relaxation time, we can derive Equation (1):

(1) $$t_{\mathrm{ind}}=t_{n}+t_{g}$$

The induction time is frequently used to measure nucleation events. Through a simplified assumption, it can be considered that temperature is inversely proportional to the nucleation rate [34]. According to classical nucleation theory [35], the relationship among the nucleation rate, induction time, and supersaturation can be expressed as Equations (2) and (3). Combining Equations (2) and (3), we derive Equation (4).

(2) $$J=\frac{K}{t_{\mathrm{ind}}}=A\exp(-\frac{\Delta G_{c}}{kT})=A\exp(-\frac{16\pi \gamma^{3}M^{2}N_{a}}{3R^{3}T^{3}\rho^{2}{\left(\ln S\right)}^{2}})$$

(3) $$t_{\mathrm{ind}}=\frac{K}{A}\exp(\frac{16\pi \gamma^{3}M^{2}N_{a}}{3R^{3}T^{3}\rho^{2}{\left(\ln S\right)}^{2}})$$

(4) $$\ln t_{\mathrm{ind}}=B+\frac{16\pi \gamma^{3}M^{2}N_{a}}{3R^{3}T^{3}\rho^{2}{\left(\ln S\right)}^{2}}$$

where *K* is a constant; *N*_a_ is the Avogadro constant, 6.023 × 10^23^ mol^−1^; *B* is a dimensionless empirical constant; *M* is the molar mass, kg/mol; *R* is the gas constant, 8.314 J K^−1^ mol^−1^; *T* is temperature, K; *ρ* is the crystal density, kg/m^3^; and *γ* is the interfacial tension between solids and he solvent.

Temperature is an important factor affecting the crystal nucleation induction time. Liu and Nancollas [36] proposed an empirical equation (Equation (5)) that reflects the relationship between the induction time *t*_ind_ and temperature:

(5) $$t_{\mathrm{ind}}=\tau \exp(E_{\mathrm{act}}/RT)$$

where *E*_act_ (J/mol) represents the activation energy of the nucleation reaction and *τ* is a constant.

### 2.2. Primary Nucleation and Parameters

#### 2.2.1. Primary Homogeneous Nucleation

Primary nucleation refers to the spontaneous formation of crystal nuclei in a solution without pre-existing crystals. According to the classical nucleation theory, the homogeneous nucleation rate equation is given as Equation (6). Δ*G*_c_ represents the critical nucleation free energy, which consists of two components: the area free energy change Δ*G*_s_, and the volume excess free energy Δ*G*_v_. When the crystal nucleus is spherical, Δ*G*_s_ and Δ*G*_v_ can be expressed as shown in Equation (6).

(6) $$\Delta G_{c}=\Delta G_{s}+\Delta G_{v}=4\pi r^{2}\gamma +\frac{4}{3}\pi r^{3}\Delta G_{\nu }$$

where Δ*G*_ν_ denotes the free energy change per unit volume during transformation. *γ* is the interfacial tension (J/m^2^), which refers to the interfacial tension between the developing crystal surface and the surrounding supersaturated solution. Δ*G*_c_ attains a maximum value Δ*G*_max_ corresponding to the critical nucleus radius *r*_c_. When the bulk free energy and interfacial free energy are balanced (dΔ*G*_c_/d*r* = 0), Δ*G*_ν_ can be calculated using Equation (7). According to the Gibbs−Thomson equation, *r*_c_ can be determined via Equation (8).

(7) $$\Delta G_{\nu }=-\frac{2\gamma }{r_{c}}$$

(8) $$r_{c}=\frac{2\gamma M}{RT\rho \ln S}$$

From the above equation, it follows that at a given temperature, the curve of ln (*t*_ind_) versus ln^−2^(*S*) follows a linear relationship, with the slope represented by α (Equation (9)). The interfacial tension γ can be calculated using Equation (10).

(9) $$\alpha =\frac{16\pi \gamma^{3}M^{2}N_{a}}{3R^{3}T^{3}\rho^{2}}$$

(10) $$\gamma =RT{\left(\frac{3\rho^{2}\alpha }{16\pi M^{2}N_{a}}\right)}^{1/3}$$

#### 2.2.2. Primary Heterogeneous Nucleation

Homogeneous nucleation is uncommon in practice due to the presence of foreign particles (such as dust and dirt) and surfaces (such as container walls) [34]. These foreign particles and surfaces reduce the excess surface energy of pre-nucleation clusters, thereby lowering the activation barrier required for nucleation and inducing heterogeneous nucleation. Under heterogeneous conditions, the total free energy change Δ*G*′_c_ required to form a critical nucleus is less than the total free energy change Δ*G*_c_ under homogeneous nucleation conditions. Δ*G*′_c_ and Δ*G*_c_ are related through Equation (11).

(11) $$\Delta {G^{\prime }}_{c}=\varphi \Delta G_{c}$$

where *φ* is the characteristic factor, defined as *φ* = *B*_het_/*B*_hom_ (the slope ratio of the linear fits for homogeneous and heterogeneous nucleation), with *φ* < 1. *φ* can be expressed as Equation (12).

(12) $$\varphi =\frac{(2+\cos\theta ){\left(1-\cos\theta \right)}^{2}}{4}$$

where *θ* represents the contact angle between the nucleus and foreign solid impurities, with 0° ≤ *θ* ≤ 180°. The contact angle reflects the affinity between the nucleus and foreign impurities, thereby determining the influence of foreign impurities on primary nucleation.

#### 2.2.3. Surface Entropy Factor

The surface entropy factor (*f*) serves as a metric for evaluating the roughness of a crystal surface and is a critical parameter in identifying crystal growth mechanisms. A higher *f* value indicates a smoother crystal surface, making crystal growth more difficult. When *f* is less than 3, rough interfaces are expected, allowing continuous crystal growth. When *f* exceeds 5, spiral growth or spiral dislocations occur, resulting in very low growth rates and highly smooth crystal surfaces. When *f* is between 3 and 5, nucleation and diffusion growth are anticipated. Therefore, the growth mechanism of a crystal can be determined based on the *f* value. For practical solution growth systems, the surface entropy factor *f* can be calculated using interfacial tension data (Equation (13)) [37].

(13) $$f=\frac{{4V_{m}}^{2/3}\gamma }{kT}$$

where *V*_m_ (*V*_m_ = *M*/*ρ*, m^3^/mol) is the volume of molecule; *k* is the Boltzmann constant (J/K).

#### 2.2.4. Key Assumptions

The critical assumptions underlying the extraction of parameters from the classical nucleation theory model include the following: (1) the shape assumption: the primary nucleus is assumed to be spherical. This is a standard simplification in classical nucleation theory, facilitating the treatment of interfacial energy by minimizing surface area. (2) Constant interfacial tension: the interfacial tension *γ* is assumed to be independent of supersaturation within the range of supersaturation considered in this study. (3) Interfacial model: for heterogeneous nucleation, we adopt the classical capillary model (hemispherical cap model). The contact angle *θ* is treated as a fitting parameter dependent on substrate properties rather than a pre-defined constant.

### 2.3. Empirical Secondary Nucleation

Secondary nucleation refers to the formation of new nuclei in the presence of existing crystals in a solution due to factors such as crystal collisions and fluid shear [34,38]. This is the most important nucleation mechanism in industrial applications and is easier to control. The nucleation systems in suspension crystallizers often differ fundamentally from those proposed by classical nucleation theory. Secondary nuclei can originate either from seed crystals or from dissolved substances near crystal surfaces. This may result from structural changes in the solution near the solid phase surface. The secondary nucleation model proposed for unseeded (clear liquor) batch crystallizers can be captured by classical power−law equations, as shown in Equation (14) [39].

(14) $$J=K_{s}S^{n}$$

where *K*_s_ is the empirical secondary nucleation rate constant, and *n* is the secondary nucleation order. Garside et al. [40] reported that the maximum value of *n* is approximately 3.

According to Equations (2) and (14), the correlation between *S* and *t*_ind_ can be described by Equation (15). The secondary nucleation parameter can be determined from the slope of the straight line between ln*t*_ind_ and ln*S*.

(15) $$\ln\;t_{\mathrm{ind}}=\ln K-n\ln S$$

### 2.4. Growth Mechanism

Crystal growth is the process by which solute molecules/ions orderly stack on the surfaces of formed nuclei, thereby increasing the crystal size. The growth rate is controlled by both diffusion (solute transfer to crystal surface) and surface reactions (solute incorporation into lattice). The initial nucleation rate and crystal growth rate determine the induction time. Generally, the induction time can be expressed as Equation (16) [41].

(16) $$t_{\mathrm{ind}}=\frac{1}{JV}+\frac{\alpha }{{\left(a_{n}JG^{n-1}\right)}^{1/n}}$$

The first part of Equation (16) assumes that the emergence of the first batch of nuclei causes the system to leave the metastable state. The loss of metastable equilibrium is attributed to the single-nucleus nucleation mechanism. The second part of Equation (16) assumes that the metastable state is lost through the statistical nucleation and growth of a large number of nuclei (multi-nucleus mechanism). Compared to the multi-nucleus mechanism, the single-nucleus mechanism is often negligible. Therefore, the induction time for the formation of the new phase via the multi-nucleus mechanism is given by Equation (17).

(17) $$t_{\mathrm{ind}}=\frac{\alpha }{{\left(a_{n}JG^{n-1}\right)}^{1/n}}$$

**Figure 1:**
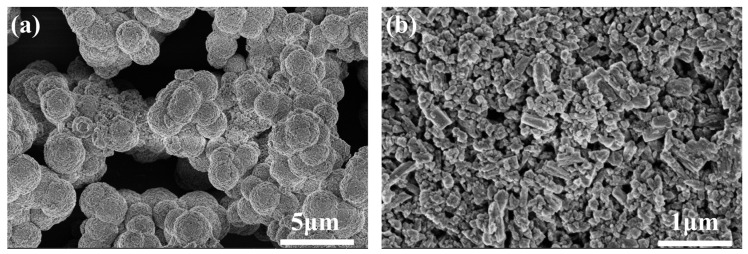
SEM images of Li_3_PO_4_ particles: (**a**) 5 μm (**b**) 1 μm.

In the formula, *a*_n_ is the shape factor, where *n* = *m*ν + 1 (*m* = 1, 2, 3 is the dimensionality of the growth; ν is a number between 0.5 and 1) and *G* is the crystal growth rate. The growth dimension of Li_3_PO_4_ crystals in this study was determined by measuring the crystal morphology. From Figure 1, it can be observed that Li_3_PO_4_ crystals aggregate into spherical shapes (Figure 1a) from flat-plate-like (Figure 1b) crystals, hence *m* = 2, which is consistent with literature reports [29]. Generally, the relationship between *G* and *S* is of the form Equation (18).

(18) $$G=K_{G}f(S)$$

In this equation, *K*_G_ is the growth rate constant, and *f* (*S*) is a given function of supersaturation, which is determined by the growth mechanism. Equation (18) can be rearranged as Equation (19).

(19) $$F(S)=\ln A_{u}+\frac{B}{n\ln^{2}S}\equiv \ln\left\{S^{1/n}{\left[f(S)\right]}^{(n-1)/n}t_{\mathrm{ind}}\right\}$$

Depending on the growth mechanisms, Equation (19) can be expressed in another form shown in Table 1. The *f* (*S*) expressions for normal, spiral, diffusion-controlled, and 2D-nucleation-mediated growth [42] are presented in Table 1. A fitting procedure can be employed to identify the growth mechanism. The best fit of *F* (*S*) vs. 1/ln^2^(*S*) characterized by the square coefficient provides information about different possible mechanisms.

## 3. Experiment

### 3.1. Materials and Methods

The lithium-containing raw solution used in this experiment is “lithium−rich brine”, which refers to the unconcentrated lithium-containing solution extracted from the Dong−Taijinaier salt lake prior to the precipitation of Li_2_CO_3_. The main ion content in lithium-rich brine is shown in Table 2 [32]. Lithium chloride anhydrous (LiCl, AR, ≥99.0%) was purchased from Tianjin Yongda Reagent Development Center (Tianjin, China), sodium phosphate dodecahydrate (Na_3_PO_4_·12H_2_O, AR, ≥98%) was purchased from Shanghai Macklin Biochemical Co., Ltd. (Shanghai, China), sodium chloride (NaCl, AR, ≥99.5%) and potassium chloride (KCl, AR, ≥99.5%) were purchased from Sinopharm Chemical Reagent Co., Ltd. (Shanghai, China), sodium sulfate (Na_2_SO_4_, AR, ≥99%) was purchased from Tianjin Guangfu Technology Development Co., Ltd. (Tianjin, China), and sodium tetraborate decahydrate (Na_2_B_4_O_7_·10H_2_O, AR, ≥99.5%) was purchased from Tianjin Hedong District Hongyan Reagent Factory (Tianjin, China). All chemical reagents used in the experiment are analytical grade reagents and do not require further purification for use. The deionized water (resistivity of 18.2 MΩ·cm at 298.15 K) used in the experiment was prepared via the ultra-pure water mechanism (Chongqing Moore Co., Ltd., Chongqing, China) in the laboratory. Weigh the samples using an analytical balance with a precision of 0.0001 g (ME204, Mettler−Toledo, Columbus, OH, USA). The pH value is measured using a pH meter with an uncertainty of ±0.01 (FiveEasy Plus, FE28, Mettler−Toledo, Columbus, OH, USA). Scanning electron microscopy (SEM, SU8010, Oxford Instruments Limited, Oxford, UK) has been used for sample imaging. The imaging was performed using the secondary electron imaging mode, with an accelerating voltage of 2.0 kV, a probe current of 13 pA, a working distance of 10.2 mm, and images captured at an appropriate resolution.

### 3.2. Experiment Setup

**Figure 2:**
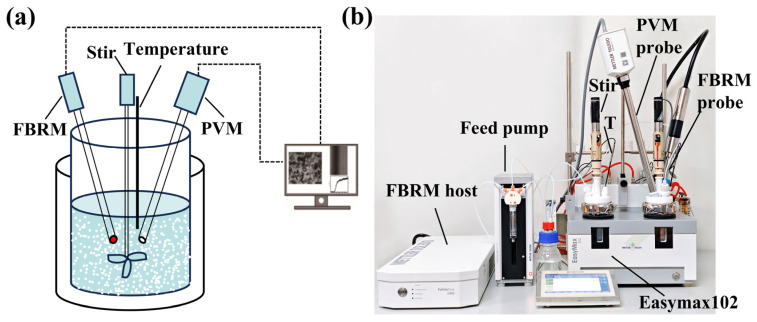
(**a**) Schematic diagram and (**b**) picture of experimental setup used for induction time, direct nucleation control, and supersaturation control experiments.

The induction time experiment was conducted using FBRM measurements, as shown in Figure 2. Simultaneously, PVM auxiliary monitoring ensured the reliability of experimental data. The relative backscatter index (RBI) in PVM serves as a sensitive process trend indicator for particle changes and can be used to determine nucleation points [43]. The measurement range of the FBRM probe is 0.5–2000 µm. FBRM employs an automatic, parameter-adaptive objective algorithm system. Adaptive Kalman filtering is used, where process noise and measurement noise parameters are automatically estimated from experimental baseline periods. Feature vectors for different particle size counts are constructed, and the Hotelling T^2^ statistic is calculated. The Bayesian online change-point-detection algorithm is applied to determine the nucleation initiation time. A sliding window model fitting with AIC-criterion monitoring is adopted, and valid fitting endpoints are identified when three consecutive window AIC values show significant increases and residuals pass the Ljung−Box white noise test (*p* < 0.05). Based on baseline data-distribution characteristics (normality test), threshold algorithms using standard deviation (kσ) or percentiles are automatically selected. The k-value is optimized to control the false positive rate (FPR < 0.01%). To obtain sufficient information during rapid nucleation, the instrument’s operating parameters are set as follows: measurement speed of 2 m/s, measurement duration of 2 s, without time averaging, without chord length weighting. The sampling rate of PVM is set to 1 frame per 2 s. Experiments were conducted in an automated synthesis reactor (Easymax102, Mettler−Toledo, Columbus, OH, USA). The Easymax102 system is equipped with a high-precision thermostatted jacket and a feedback-controlled heating/cooling system. It actively compensates for any temperature fluctuations, including those generated by the exothermic heat of crystallization, by adjusting the jacket temperature in real-time. This ensures that the solution bulk temperature remains isothermal (±0.1 K) throughout the experiment. The crystallizer was a 100 mL glass reactor with an integrated top-mounted stirrer, and the entire experiment was temperature-controlled at ±0.1 K.

### 3.3. Induction Time Measurements

**Figure 3:**
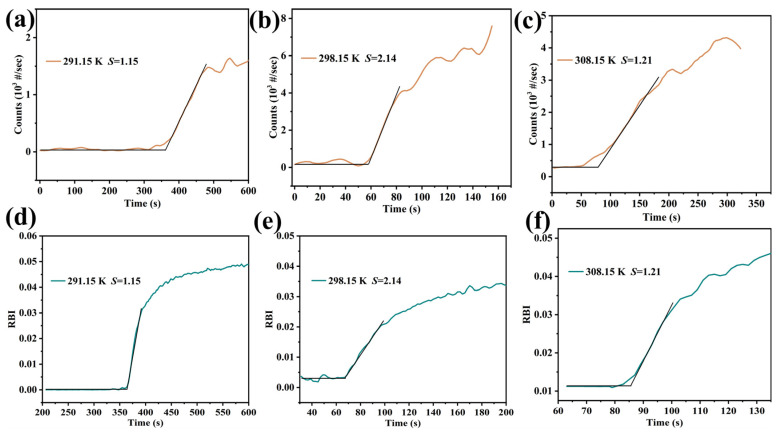
Time-dependent variation of particle number or RBI values measured at (**a**,**d**) *T* = 291.15 K, *S* = 1.15, (**b**,**e**) *T* = 298.15 K, *S* = 2.14, (**c**,**f**) *T* = 308.15 K, *S* = 1.21. The black lines in the figure represents the linear regression of the chord number or RBI over time in two different regions.

This study employed FBRM and PVM to determine the induction time of Li_3_PO_4_ crystallization. First, 50 mL of a lithium-rich brine or LiCl solution was placed into the crystallizer. The pH of the solution was then adjusted to 11.0 using a 10 mol·L^−1^ NaOH solution. This will ensure that the phosphate ions in the solution primarily exist in the PO_4_^3−^ form, reducing the complexity of the coexistence of multiple hydrogen phosphate ions [18]. The FBRM and PVM probes were immersed in the solution adjacent to the agitator. The temperature and stirring speed (400 rpm) were set using the Easymax102 system software (iControl, version 6.0), and the process was initiated. After the solution temperature stabilized, the FBRM and PVM were activated. A predetermined volume of a 0.4 mol·L^−1^ Na_3_PO_4_ solution was then rapidly injected into the crystallizer (10 mL/min). Use a propeller stirrer to feed material at the position directly above the impeller blade. This location resides in the region within the reactor vessel where shear forces and convection are most intense, facilitating instantaneous dispersion of the feed material and ensuring thorough mixing of the components. The onset of burst nucleation was identified by a sharp increase in the total chord length, as monitored via FBRM (Figure 3). The nucleation onset point was determined by applying linear regression to two distinct regions of the chord count or RBI versus time plot and identifying their intersection. The time interval between the initial injection of the Na_3_PO_4_ solution and this nucleation onset was recorded as the induction time. The reaction between the lithium-rich brine and Na_3_PO_4_ is rapid, typically completing within minutes. Consequently, the reaction time was considered negligible in the induction time calculations owing to the fast kinetics [44]. Representative plots obtained at various supersaturation levels and temperatures are shown in Figure 3. As illustrated in Figure 3, the induction time decreases markedly with increasing supersaturation or temperature.

All the kinetic parameters obtained in this study were determined under the specific condition of pH 11 regulated by NaOH. Due to the introduction of Na^+^, the ionic strength of the system was increased. Therefore, the nucleation kinetic parameters derived from induction period data should be regarded as apparent parameters under this particular ionic environment. Although the presence of Na^+^ might subtly affect the nucleation process through electrostatic shielding or complexation effects, we believe that the fundamental trends and qualitative mechanisms revealed in this study regarding induction period variations with supersaturation, temperature, etc., remain reliable and valid.

## 4. Results and Discussion

### 4.1. Induction Time

As a fundamental parameter in nucleation research, the *t*_ind_ connects the nucleation theory with experimental observations. Utilizing the nucleation theory, the relationship between the *t*_ind_ and supersaturation was investigated. In this work, supersaturation is determined by the concentration and solubility of Li_3_PO_4_. The solubility of Li_3_PO_4_ in pure water and lithium-rich brine has been studied [32]. The solubility of Li_3_PO_4_ in lithium-rich brine (Table 3) will be used to calculate supersaturation *S*.

Simultaneously detect the induction time with both FBRM and PVM instruments to investigate the sensitivity of the detection method. By comparison, the sensitivity of the induction time detected by the PVM instrument lags behind that of the FBRM by approximately 5–10 s, while induction-time trends measured by the two test devices are the same (Figure 3). The experimental results verified the sensitivity of the FBRM instrument. Therefore, the results from the PVM instrument were used to standardize the nucleation-induction time data. All experiments were repeated at least three times, and the average results were reported.

#### 4.1.1. Impact of Temperature and Supersaturation

The relationship between the induction time and supersaturation can generally be described by empirical Equation (20).

(20) $$t_{\mathrm{ind}}=\frac{K_{1}}{S^{\delta }}$$

where *K*_1_ and *δ* are empirical constants.

**Figure 4:**
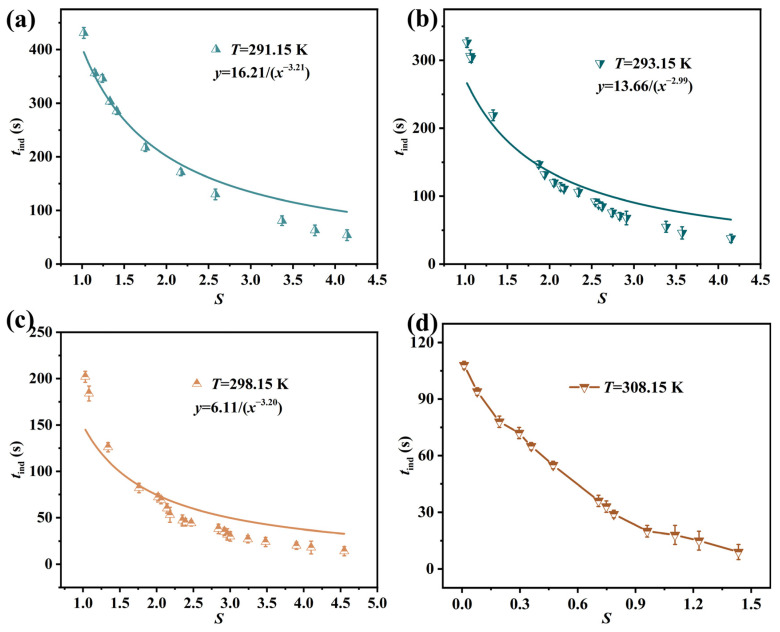
Relationship between induction time *t*_ind_ and supersaturation *S* of Li_3_PO_4_ at different temperatures: (**a**) *T* = 291.15 K (**b**) *T* = 293.15 K (**c**) *T* = 298.15 K (**d**) *T* = 308.15 K. The solid lines in (**a**–**c**) represent the fitting results of the empirical equation (Equation (20)).

According to the crystallization theory, supersaturation is a primary factor affecting the nucleation rate and induction time. The induction time curves under different supersaturations in the temperature range of 291.15 K−308.15 K are shown in Figure 4. It can be seen that the induction time decreases with increasing supersaturation and temperature. When the supersaturation gradually increases and enters the high-supersaturation region (*S* > 2.5), the curve becomes flat. Nucleation in the solution results from solute molecules reaching a critical cluster size through motion and collisions. The increase in temperature or supersaturation promotes solute molecular motion and collisions, thereby increasing the nucleation rate [22,45]. Since the induction time is inversely proportional to the nucleation rate (Equation (2)), the nucleation rate increases with supersaturation. The induction time and supersaturation under different temperatures can be related based on Equation (20), with the parameters obtained as listed in Table 4. It can be observed that the correlation coefficient R^2^ decreases with an increasing temperature. When the temperature is 291.15 K, the R^2^ value of the empirical equation fitting is 0.9526. From the Figure 4, it can be observed that the deviation of the fitted empirical equation increases with increasing supersaturation. This may be attributed to the fact that under high-supersaturation conditions, the crystal nuclei growth strongly depends on the supersaturation level, rendering the growth time no longer negligible. Consequently, the induction time deviates from the classical nucleation theory curves based on steady-state and pure nucleation processes, with the deviation systematically increasing as supersaturation rises. This phenomenon also reveals the limitations of applying classical nucleation theory to study the Li_3_PO_4_ reaction crystallization process, as well as the complexity of nucleation mechanisms.

#### 4.1.2. Effect of Ultrasound

**Figure 5:**
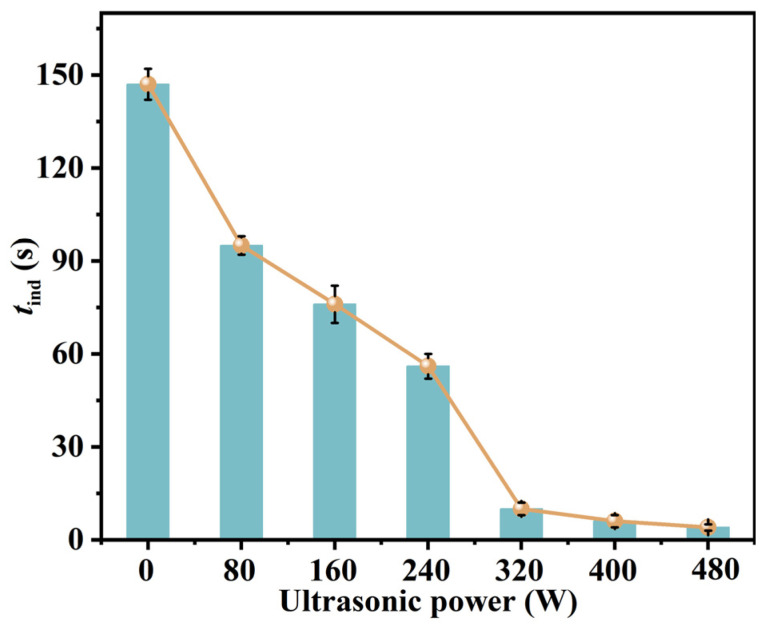
Effect of ultrasonic power on induction time *t*_ind_ (*T* = 293.15 K, supersaturation *S* = 1.87, ultrasonic frequency: 40 kHz, power density: 0–9.6 W/mL, duty cycle: 33%).

The ultrasonic probe (FANGXU, L−400Z, Shanghai Fang Xu Technology Co., Ltd., Shanghai, China) was inserted into the solution to measure the effect of ultrasonic power on the induction time. The ultrasonic burst cycle was set to 1 s with 2 s intervals between bursts. The induction time across the ultrasonic power range of 0−480 W is shown in Figure 5. It can be observed that as the ultrasonic power increases, the induction time decreases, indicating that ultrasound shortens the induction time and facilitates nucleation. This is because the alternating vibration of cavitation bubbles and bubble collapse generates pressure that increases the diffusion coefficient, while the hotspots induced by ultrasound enhance supersaturation in certain micro-regions, thereby accelerating the nucleation process.

#### 4.1.3. Impacts of Impurities

In the industrial crystallization process, even low-concentration impurities can significantly alter the crystallization process. Impurities can affect the nucleation and growth rates of crystals, alter the properties of the solution, modify the adsorption-layer characteristics at the crystal−solution interface, and influence the integration of growth units. They can be incorporated into the crystal, particularly when there is a degree of lattice similarity [34]. Therefore, the induction time has an inherent relationship with the initial nucleation rate. If an impurity can inhibit the initial nucleation rate, the induction time must be extended.

**Figure 6:**
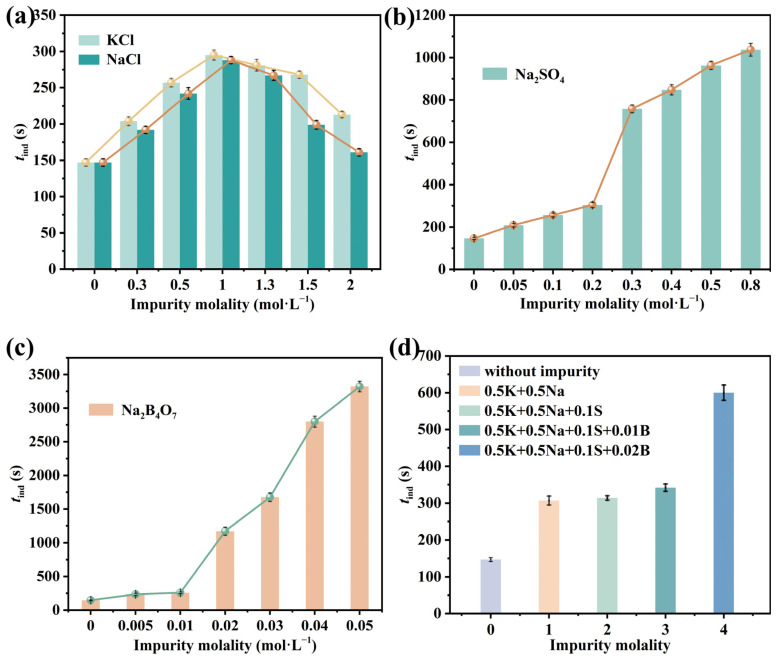
Effect of impurities on induction time *t*_ind_ (*T* = 293.15 K and supersaturation *S* = 1.87). (**a**) KCl, NaCl salt solution; (**b**) Na_2_SO_4_ salt solution; (**c**) Na_2_B_4_O_7_ salt solution; (**d**) KCl-NaCl-Na_2_SO_4_^−^Na_2_B_4_O_7_ mixed salt solution.

KCl, NaCl, Na_2_SO_4_, and Na_2_B_4_O_7_ are the main companion components in lithium-rich brine from the Dong−Taijinaier salt lake, so they were first selected as impurities in this study. The induction time curves of Li_3_PO_4_ in single salt solutions of KCl (0–2 mol·L^−1^), NaCl (0–2 mol·L^−1^), Na_2_SO_4_ (0–0.8 mol·L^−1^), and Na_2_B_4_O_7_ (0–0.05 mol·L^−1^) at 293.15 K are shown in Figure 6a−c. It can be observed that both Na_2_SO_4_ and Na_2_B_4_O_7_ can prolong the induction time, with a small amount of Na_2_B_4_O_7_ significantly extending it. This may be due to SO_4_^2−^ or B_4_O_7_^2−^ forming ion pairs with Li^+^, thereby reducing Li^+^ activity [46,47]. This conclusion aligns with experimental measurements of Li^+^ activity in the presence of SO_4_^2−^ or B_4_O_7_^2−^. The curves for KCl and NaCl nearly overlap, both causing an initial slight increase followed by a slight decrease. This is because, as the concentration of NaCl or KCl solutions increases, free hydrated ions, solvent−split ion pairs, contact ion pairs, and more complex ion-associated structures are formed in sequence, and these solution structures affect Li^+^ activity.

The induction time curves of Li_3_PO_4_ in mixed salt solutions (0.5 K-0.5 Na, 0.5 K-0.5 Na-0.1 S, 0.5 K-0.5 Na-0.1 S-0.01 B, and 0.5 K-0.5 Na-0.1 S-0.02 B) (the composition is as shown in the Table 5) are shown in Figure 6d. It can be observed that both Na_2_SO_4_ and Na_2_B_4_O_7_ can prolong the induction time, whereas Na_2_B_4_O_7_ exerts a very strong prolonging effect, indicating that Na_2_B_4_O_7_ exhibits the strongest nucleation inhibition effect on Li_3_PO_4_.

### 4.2. Nucleation Kinetic Mechanism and Parameters

Induction time and supersaturation regression are widely used to study the nucleation mechanisms of crystals, particularly for inorganic compounds [48,49,50]. Experimental induction time *t*_ind_ and supersaturation *S* data were analyzed using the classical nucleation theory model Equation (4) and the secondary nucleation model Equation (15).

#### 4.2.1. Classical Nucleation Mechanism and Parameters

**Figure 7:**
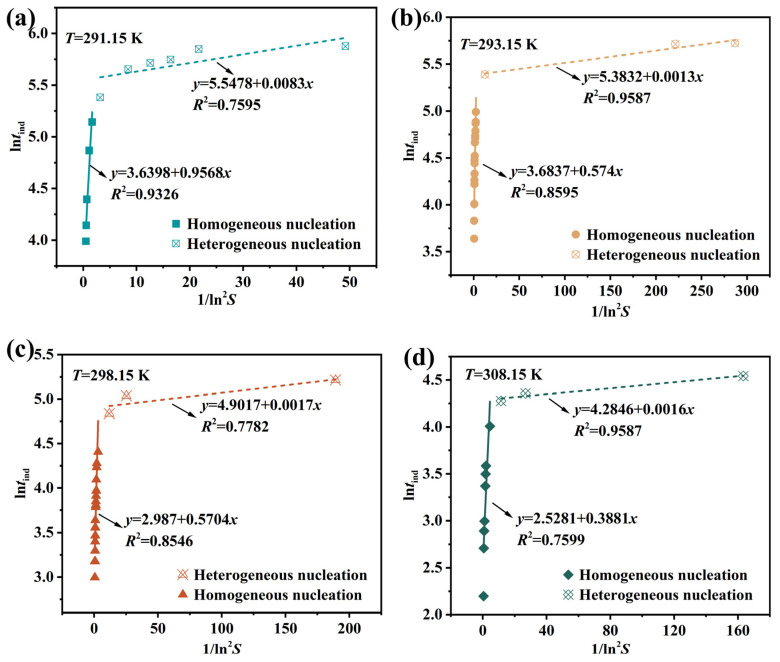
The plots of ln*t*_ind_ versus 1/ln^2^*S* at different temperatures. (**a**) 291.15 K; (**b**) 293.15 K; (**c**) 298.15 K; (**d**) 308.15 K.

Figure 7 illustrates the relationship between homogeneous and heterogeneous nucleation in Li_3_PO_4_, consisting of two straight lines with different slopes. The change in the slope indicates two distinct nucleation mechanisms: the line with the larger slope corresponds to homogeneous nucleation occurring at high supersaturation levels, while the line with the smaller slope corresponds to heterogeneous nucleation occurring at low supersaturation levels [50,51]. Under higher supersaturation conditions, homogeneous nucleation plays a significantly more dominant role than heterogeneous nucleation. At lower supersaturation levels, the phase transformation driving force is weaker, making the nucleation process more susceptible to external particles, hence heterogeneous nucleation mechanisms dominate the nucleation process. At higher supersaturation levels, the phase transformation driving force is stronger. Compared to solution spontaneous nucleation, external particle effects on the nucleation process become negligible, resulting in homogeneous nucleation mechanisms dominating the nucleation process.

Mersmann et al. [52] proposed that for the reactive crystallization of insoluble substances, the transition supersaturation (*S*_t_) required for homogeneous nucleation should exceed 2. For the reactive crystallization of Li_3_PO_4_ in our study, *S*_t_ is not a fixed value. As shown in Figure 7, at temperatures ranging from 291.15 K to 308.15 K, the corresponding *S*_t_ values are 2.17, 2.05, 2.03, and 2.02. This indicates that rising temperatures weaken the role of heterogeneous nucleation, making homogeneous nucleation relatively more likely to occur compared to heterogeneous nucleation. Therefore, Li_3_PO_4_ crystallization can be controlled at room temperature through seeding strategies.

**Figure 8:**
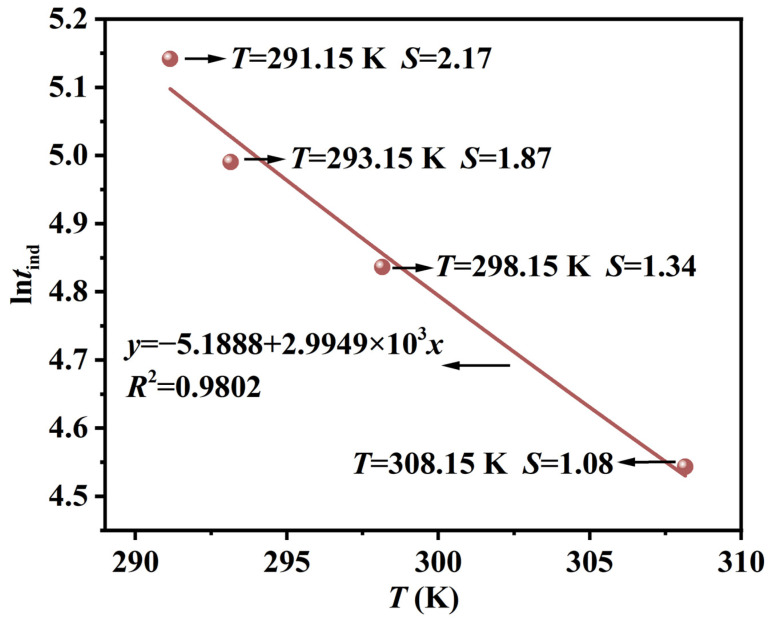
The plots of ln*t*_ind_ versus 1/*T* at different temperatures.

Figure 8 shows the linear relationship between ln*t*_ind_ and 1/*T* under four different supersaturation levels. Based on the slope of the fitted line according to Equation (5), the average activation energy *E*_act_ of Li_3_PO_4_ below 308.15 K is obtained as 24.90 kJ·mol^−1^. Due to the presence of numerous impurities in the lithium-rich brine, which lower the nucleation energy barrier, the *E*_act_ is lower.

Table 6 shows the slopes of the two straight lines obtained from Equation (4) for homogeneous and heterogeneous nucleation, along with the corresponding interfacial energy *γ* values (Equation (10)). The *γ* value can serve as an indicator of the solute self-nucleation capability, with higher values indicating greater difficulty in self-nucleation. From Table 6, the estimated ranges of interfacial energy for homogeneous and heterogeneous nucleation are 65.2–83.2 mJ·m^−2^ and 10.5–17.1 mJ·m^−2^, respectively. The *γ* (homogeneous nucleation) value aligns with the surface tensions of weakly soluble inorganic salts like CaHPO_4_·2H_2_O, CaSO_4_·2H_2_O, and CaCO_3_ within the 68–97 mJ·m^−2^ range [29,53]. Lower interfacial energy indicates easier solute crystallization from a solution. Notably, the interfacial energy for heterogeneous nucleation is lower than that for homogeneous nucleation, consistent with our expectations. It decreases slightly with an increasing temperature, suggesting easier nucleation at higher temperatures. In other words, temperature elevation favors nucleus formation. Although some data points exhibit minor fluctuations due to deviations in the fitted straight line slopes in experimental data, this does not affect the overall trend. At 291.15 K, the contact angle *θ* is 27.23°, higher than values at other elevated temperatures, indicating that the system predominantly undergoes heterogeneous nucleation.

**Figure 9:**
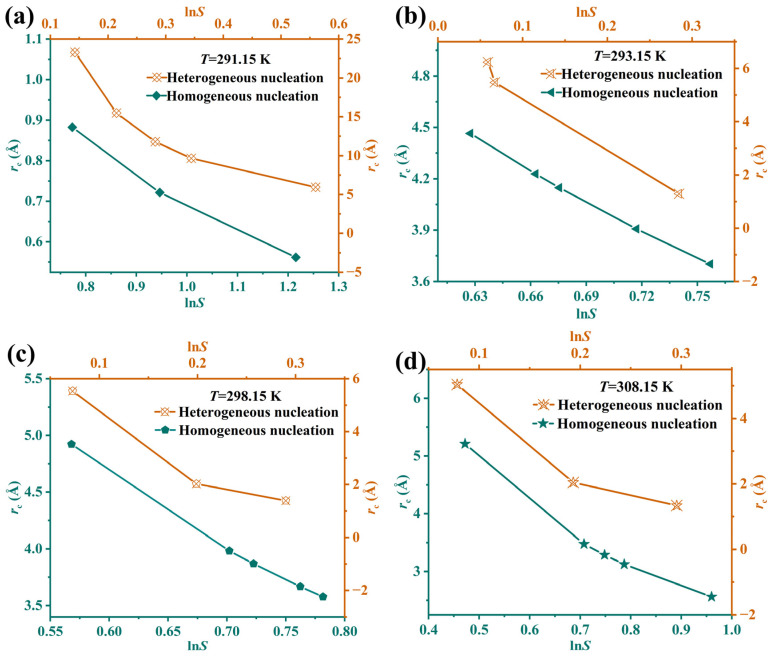
Variation of critical nucleus size *r*_c_ with supersaturation under different temperatures. (**a**) 291.15 K; (**b**) 293.15 K; (**c**) 298.15 K; (**d**) 308.15 K.

The surface entropy factor *f* values calculated from Equation (13) are shown in Table 6. It can be observed that *f* decreases with an increasing temperature. This indicates that as the temperature rises, the energy barrier for Li_3_PO_4_ growth in lithium-rich brine becomes lower, allowing continuous crystal growth of Li_3_PO_4_ nuclei. When *f* > 5, it is preliminarily inferred that Li_3_PO_4_ exhibits a spiral growth mechanism in lithium-rich brine. The obtained *f* values in this study are consistent with reported surface entropy factors of Li_2_CO_3_ [54]. According to classical nucleation theory, substituting *γ* into Equation (8) yields the critical nucleation size *r*_c_. It can be seen from Figure 9 and Table 7 that the *r*_c_ value decreases as the supersaturation *S* or temperature increases. The *r*_c_ range (1–23 Å) falls within the same magnitude as other model substances (8–25 Å) [55]. The critical nucleation size for heterogeneous nucleation is smaller than that for homogeneous nucleation. It can be seen that the critical nucleus size is extremely small (typically ranging from several to dozens of molecules), indicating that the nucleation process is essentially a process where molecular clusters reach a stable size, rather than a macroscopic phase transition. The rate at which reaction products are generated determines the rate of supersaturation formation, serving as the key factor in controlling nucleation.

These results further verify that both the supersaturation and temperature jointly control the nucleation process. Therefore, increasing the supersaturation or temperature can enhance the nucleation rate and shorten the induction time.

#### 4.2.2. Secondary Nucleation Mechanism and Parameters

**Figure 10:**
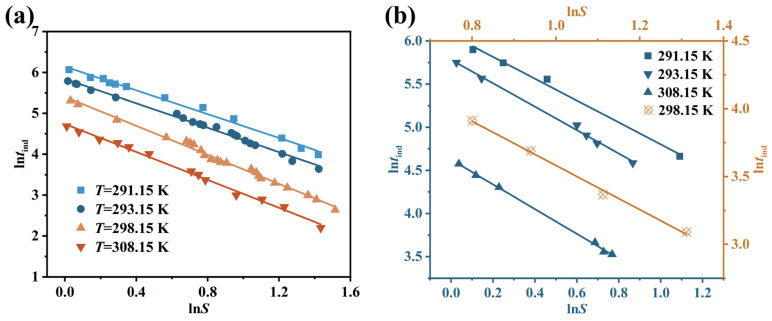
Dependence of ln*t*_ind_ on ln*S* under the secondary nucleation model for Li_3_PO_4_ at different temperatures. (**a**) Linear regression of ln*t*_ind_ versus ln*S*; (**b**) Comparison of experimental and predicted induction times.

As shown in Figure 7 and Figure 10a, linear regression was performed on ln*t*_ind_ versus 1/ln^2^*S* and ln*t*_ind_ versus ln*S* at different temperatures to determine the primary nucleation mechanism of Li_3_PO_4_. It can be inferred that the induction time of Li_3_PO_4_ crystallization better fits the linear relationship described by the secondary nucleation model (Equation (15)). The correlation indices of both models at different temperatures are shown in Table 8. The secondary nucleation model governs the nucleation process because its correlation coefficient is closer to 1. The slope (*n*) and intercept of the secondary nucleation model (Equation (15)) at different temperatures are presented in Table 8. At higher temperatures, increased molecular diffusion enhances the nucleation rate, resulting in an upward trend of the nucleation order (*n*) with a temperature rise. Figure 10b compares the experimental induction time and predicted induction time of Li_3_PO_4_ under the secondary nucleation model at different temperatures. It can be observed that the correlation is strong, demonstrating that the secondary nucleation model is accurate and reliable.

The determination of nucleation mechanisms is crucial because the product specifications of batch crystallization are initially controlled by nucleation [56]. In this study, no seed crystals were added at the start of the operation, so primary nucleation precedes secondary nucleation. It can be inferred that only a few crystals are formed via primary nucleation, followed by secondary nucleation. In other words, these newly formed crystals gradually grow and assume the role of new seed crystals, creating the next generation of crystals [33]. The relevant indicators in Table 8 confirm that secondary nucleation is more significant than primary nucleation. The non-crystalline absorption layer of the solute (a boundary layer near the growing crystals formed by initial nuclei) serves as a site for secondary nucleation in non-seeded crystallization. Higher supersaturation thickens the absorption layer and generates numerous nuclei. Greater supersaturation reduces the critical nucleus size (Table 7), thereby increasing the likelihood of continuous nucleation. By increasing supersaturation, the number of atomic nuclei increases, enhancing the probability of sustained nucleation and consequently shortening the induction time (Figure 10) [33].

### 4.3. Growth Kinetics Mechanism and Parameters

**Figure 11:**
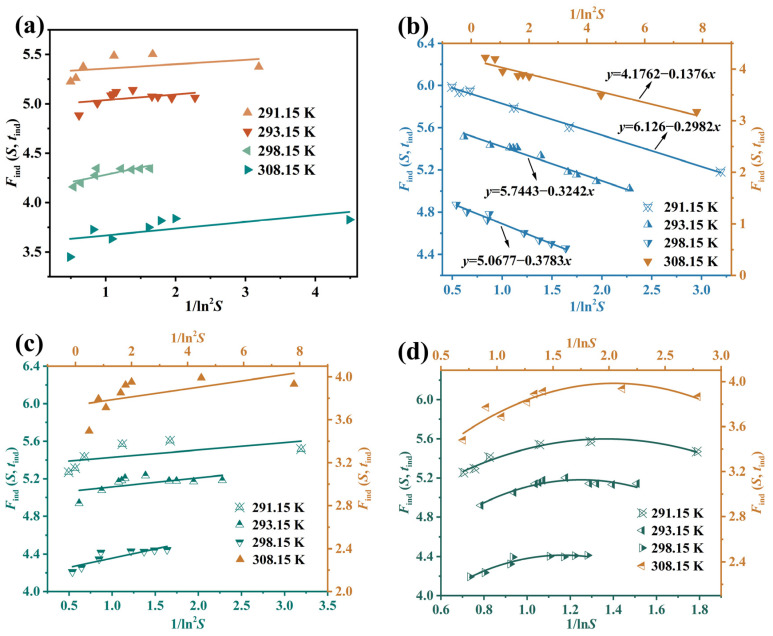
The relationship between *F*_ind_ (*S*, *t*_ind_) for Li_3_PO_4_ and 1/ln^2^*S* or 1/ln*S*. Data fitting using (**a**) normal growth, (**b**) spiral growth, (**c**) diffusion-controlled growth, and (**d**) 2D-nucleation-mediated growth.

Figure 11 shows the fitted results of seedless crystallization experiments under different supersaturations to determine the growth mechanism of Li_3_PO_4_. As shown in Figure 11b,d and Table 9, the fitted curves of the spiral growth mechanism and 2D nucleation-mediated model are consistent with the experimental results. Simultaneously, combining with the aforementioned surface entropy factor (*f* > 5), it confirms that the growth mechanism of Li_3_PO_4_ is more consistent with the spiral growth. From this, we can deduce that the active sites for Li_3_PO_4_ crystal growth originate from internal defects (dislocations) rather than crystal faces. The edges of spiral steps serve as high-energy active sites for impurity adsorption. Trace impurities readily adsorb onto these steps, blocking their advancement and significantly altering growth rates and crystal habit. Since spiral growth is effective under low supersaturation levels, the process design should prioritize the precise control of supersaturation rather than pursuing high supersaturation to drive growth. Steady spiral growth facilitates ordered molecular incorporation into the lattice, reducing solute or impurity entrapment caused by excessive growth rates, thereby improving product purity and stability. In reactive crystallization, strict control of the feed rate and mixing efficiency is essential to regulate the peak of instantaneous supersaturation. Therefore, for Li_3_PO_4_ reactive crystallization, low and stable supersaturation should be pursued, precise control of impurities should be focused on, and seed crystals should be utilized to achieve controlled growth.

### 4.4. Online Monitoring of Li_3_PO_4_ Crystallization Process

**Figure 12:**
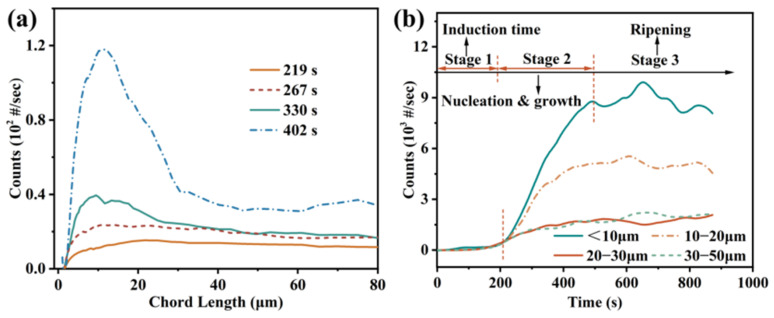
Online monitoring of the Li_3_PO_4_-crystallization process; (**a**) evolution of particle-size distribution over time; (**b**) time evolution of particle counts across different size intervals.

Figure 12 shows the experimental results of the typical Li_3_PO_4_-nucleation process (293.15 K, 400 rpm, *S* = 1.33). The division into different stages of the Li_3_PO_4_-crystallization process is primarily based on the real-time tracking of the particle count and particle size distribution using FBRM to infer the dominant mechanisms at each stage. Figure 12a displays the time-dependent evolution of the particle-size distribution. After a period of induction, the grain size-distribution rapidly increased and shifted to the right, indicating the nucleation and growth processes of crystals. Figure 12b depicts the temporal evolution of particle counts across distinct size intervals. At the onset of Stage 2, the population of small particles (<10 μm) exhibits a sharp, rapid increase. This surge is characteristic of explosive primary nucleation, indicating the massive formation of new particles. Subsequently, the stabilization of particle counts in the latter part of Stage 2 suggests that crystal growth becomes the dominant process. Thus, although nucleation and growth coexist during Stage 2, their dominant mechanisms undergo a transition. As shown in Figure 12b, the crystallization process can be divided into three stages. The first stage represents the induction phase. The second stage primarily involves crystal nucleation and growth. Agglomeration and breakage also occur to some extent, with supersaturation mainly consumed during this phase. In Phase 3, the supersaturation decreases to a relatively low level, where crystal nucleation and growth continue but are not prominent, gradually transitioning the crystallization process into the maturation phase. In Li_3_PO_4_ reactive crystallization, the real-time monitoring of supersaturation or the particle count can immediately detect abnormal nucleation, growth, or agglomeration/fragmentation, allowing for the timely adjustment of parameters (e.g., feed rate, stirring) to ensure that the target particle size is achieved.

## 5. Conclusions and Limitations

### 5.1. Conclusions

To precisely control the reactive crystallization process of Li_3_PO_4_, the induction time was determined using FBRM, and the effects of temperature, supersaturation, impurities, and ultrasound on the induction time were investigated. The primary nucleation and growth kinetic mechanisms for extracting Li_3_PO_4_ from low-concentration lithium-rich brine were evaluated using the induction time method, along with the regression of kinetic parameters. Monitor the particle counts and particle size distribution in real-time during the reaction crystallization process of Li_3_PO_4_. The results showed that the induction time decreased with an increasing temperature/supersaturation/ultrasound frequency, indicating that their elevations all promote nucleation. Impurities (NaCl/KCl) did not significantly affect the induction time, whereas Na_2_SO_4_ and Na_2_B_4_O_7_ significantly increased it, with Na_2_B_4_O_7_ showing the most notable effect. This indicates that Na_2_SO_4_ and Na_2_B_4_O_7_ inhibit nucleation, with Na_2_B_4_O_7_ exhibiting the strongest inhibitory effect.

Based on the fitting results from the classical nucleation theory, it can be inferred that the increase in temperature weakens the effect of heterogeneous nucleation, making homogeneous nucleation relatively more favorable compared to heterogeneous nucleation. The average *E*_act_ below 308.15 K is 24.9 kJ·mol^−1^, indicating that the nucleation of Li_3_PO_4_ primarily follows a heterogeneous mechanism. The estimated interfacial energy ranges for homogeneous and heterogeneous nucleation are 65.2–83.2 mJ·m^−2^ and 10.5–17.1 mJ·m^−2^ respectively, both decreasing with temperature. This indicates that the energy barrier for heterogeneous nucleation is significantly lower than that for homogeneous nucleation, making heterogeneous nucleation more prevalent in practical systems. The surface entropy factor f > 5 decreases with an increasing temperature, indicating that crystal growth may follow a spiral growth mechanism, and higher temperatures are favorable for crystal growth. The critical nucleus size of 1–23 Å at 291.15 K−308.15 K decreases with increasing supersaturation, demonstrating that nucleation essentially involves molecular clusters reaching stable dimensions rather than macroscopic phase transitions. The contact angle *θ* of 27.23° at 291.15 K, higher than at elevated temperatures, confirms dominant heterogeneous nucleation.

The fitting results from the classical nucleation theory and empirical secondary nucleation theory model indicate that Li_3_PO_4_ predominantly follows a secondary nucleation mechanism, with a nucleation order of 1.45–1.71 that increases with temperature. By linking induction times to various growth mechanisms, the spiral growth pattern of Li_3_PO_4_ crystals was revealed. Additionally, the real-time monitoring of particle counts and particle size distribution during the crystallization process of Li_3_PO_4_ can infer the dominant mechanisms at different stages of crystallization. For industrial processes producing Li_3_PO_4_ from lithium-rich brines, we recommend synergistically controlling nucleation/growth rates. This requires the application of strategies such as fluid mechanics (stirring), thermodynamics (supersaturation, temperature), external field enhancement (supercritical gravity, ultrasonic), and seeding, combined with online monitoring (FBRM, PVM) and feedback control, to achieve precise the customization of products. These findings enhance the understanding of Li_3_PO_4_ reactive crystallization processes and are critical for designing and controlling crystallization systems to produce high-quality Li_3_PO_4_ from lithium-rich brines.

### 5.2. Limitations

This study employed FBRM online monitoring technology to investigate the nucleation and growth kinetics of Li_3_PO_4_ reaction crystallization using the induction period method. The research system was a lithium-rich brine, operating within a temperature range of 291.15–308.15 K and supersaturation range of 1.02–4.5.

It should be noted that the FBRM string length distribution does not directly equate to the particle-size distribution, and measurement results are influenced by particle morphology and local mixing conditions. Kinetic parameter extraction relies on selected models (classical nucleation theory, empirical secondary nucleation model), whose inherent assumptions may not fully reflect complex crystallization behaviors. The obtained kinetic parameters are applicable to operational conditions within this experimental range and hold certain reference value in similar systems. However, for other crystallization systems or different reactor scales, it is recommended to validate through offline analysis (e.g., microscopy, particle size analyzer), while considering factors such as the solution system, impurity effects, and equipment differences during extrapolation.

## Figures and Tables

**Table 1:** Expressions for *F*_ind_ (*S*, *t*_ind_) and *f* (*S*) corresponding to different growth mechanisms, along with possible values of υ and *n* (for *m* = 2).

Growth Mechanisms	*υ*	*n*	*F*_ind_ (*S*, *t*_ind_)	*f* (*S*)
normal growth	1	3	ln(*S*^1/3^(*S* − 1)^2/3^*t*_ind_) = ln*A*_ind_ + *B*/(3 ln^2^*S*)	*S* − 1
spiral growth	1	3	ln(*S*^1/3^(*S* − 1)^3/4^*t*_ind_) = ln*A*_ind_ + *B*/(3 ln^2^*S*)	(*S* − 1)^2^
diffusion−controlled growth	1/2	2	ln(*S*^1/2^(*S* − 1)^1/2^*t*_ind_) = ln*A*_ind_ + *B*/(2 ln^2^*S*)	*S* − 1
2D nucleation−mediated growth	1	3	ln(*S*^4/9^(*S* − 1)^5/9^*t*_ind_) = ln*A*_ind_ + 2*B*_2D_/9ln*S* +*B*/3ln^2^*S*	(*S* − 1)^2/3^*S*^1/3^exp(−*B*_2D_/3ln*S*)

**Table 2:** Main ion concentrations and pH values in lithium-rich brine.

Ions/pH	Li^+^	Na^+^	K^+^	B_2_O_3_	Cl^−^	SO_4_^2−^	pH
*c* (mg·L^−1^)	8912	9108	713	174	59,730	36.52	7.95 (at 293.15 K)

**Table 3:** Solubility data of Li_3_PO_4_ in lithium-rich brine.

*T* (K)	291.15	293.15	298.15	308.15
c* (10^−3^ mol·L^−1^)	8.790	8.766	8.718	8.670

**Table 4:** Empirical parameters obtained from the Equation (20) at different temperatures.

*T* (K)	*K*_1_	*δ*	*R* ^2^
291.15	16.21	−3.21	0.9526
293.15	13.66	−2.99	0.9068
298.15	6.11	−3.20	0.8150

**Table 5:** The content of individual components in a mixed salt solution.

Mixed Salt Solutions	Salt Solution Concentration (mol·L^−1^)			
	KCl	NaCl	Na_2_SO_4_	Na_2_B_4_O_7_
0.5 K-0.5 Na	0.5	0.5	−	−
0.5 K-0.5 Na-0.1 S	0.5	0.5	0.1	−
0.5 K-0.5 Na-0.1 S-0.01 B	0.5	0.5	0.1	0.01
0.5 K-0.5 Na-0.1 S-0.01 B	0.5	0.5	0.1	0.02

**Table 6:** The parameters of classical nucleation (Equation (4)) at different temperatures.

*T* (K)	Homogeneous Nucleation					Heterogeneous Nucleation				*θ* (°)
	Slope	Intercept	*R* ^2^	*γ* (mJ·m^−2^)	*f*	Slope	Intercept	*R* ^2^	*γ* (mJ·m^−2^)	
291.15	0.9568	3.6398	0.9326	83.2	15.16	0.0083	5.6612	0.7595	17.1	27.23
293.15	0.5740	3.6837	0.8595	70.7	12.79	0.0013	5.3832	0.9587	9.28	19.45
298.15	0.5704	2.9870	0.8546	71.7	12.76	0.0017	4.9017	0.7782	10.3	20.72
308.15	0.3881	2.5281	0.7599	65.2	11.23	0.0016	4.2846	0.9578	10.5	22.42

**Table 7:** Critical nucleation sizes *r*_c_ (Å) calculated according to Equation (8).

**Homogeneous Nucleation**							
**291.15 K**		**293.15 K**		**298.15 K**		**308.15 K**	
**ln*S***	***r*** **_c_**	**ln*S***	***r*** **_c_**	**ln*S***	***r*** **_c_**	**ln*S***	***r*** **_c_**
0.77	0.88	0.63	4.46	0.57	4.92	0.47	5.21
0.95	0.72	0.66	4.23	0.70	3.98	0.71	3.48
1.22	0.56	0.68	4.15	0.72	3.87	0.75	3.29
−	−	0.72	3.91	0.76	3.67	0.79	3.12
−	−	0.76	3.70	0.78	3.58	0.96	2.56
**Heterogeneous Nucleation**							
**291.15 K**		**293.15 K**		**298.15 K**		**308.15 K**	
**ln*S***	***r*** **_c_**	**ln*S***	***r*** **_c_**	**ln*S***	***r*** **_c_**	**ln*S***	***r*** **_c_**
0.14	23.30	0.059	6.23	0.073	5.53	0.078	5.04
0.21	15.47	0.067	5.47	0.20	2.02	0.19	2.04
0.28	11.79	0.29	1.29	0.29	1.39	0.30	1.33
0.34	9.65	−	−	−	−	−	−
0.56	5.93	−	−	−	−	−	−

**Table 8:** The determination coefficients *R*^2^ and parameters for different nucleation mechanisms of Li_3_PO_4_ at various temperatures.

*T* (K)	Classical Nucleation (Equation (4))	Secondary Nucleation (Equation (15))		
	*R* ^2^	*R* ^2^	Nucleation Order *n*	Intercept
291.15	0.9326	0.9914	1.45	6.1424
293.15	0.8595	0.9928	1.49	5.8413
298.15	0.8546	0.9918	1.77	5.4032
308.15	0.7599	0.9934	1.71	4.7381

**Table 9:** The coefficients of determination *R*^2^ for different growth mechanisms of Li_3_PO_4_ under different temperatures.

*T* (K)	Normal Growth	Spiral Growth	Diffusion—Controlled Growth	2D Nucleation—Mediated Growth
	*R* ^2^	*R* ^2^	*R* ^2^	*R* ^2^
291.15	0.1565	0.9964	0.3472	0.9651
293.15	0.1707	0.9715	0.3468	0.8739
298.15	0.6576	0.9809	0.7560	0.9349
308.15	0.4199	0.9349	0.3273	0.8648

## Data Availability

The original contributions presented in this study are included in the article. Further inquiries can be directed to the corresponding authors.
